# Crowdsourcing the Robin Hood effect in cities

**DOI:** 10.1007/s41109-017-0026-3

**Published:** 2017-06-08

**Authors:** Thomas Louail, Maxime Lenormand, Juan Murillo Arias, José J. Ramasco

**Affiliations:** 1 CNRS, UMR Géographie-cités, 13 rue du four, Paris, FR-75006 France; 2 Irstea, UMR TETIS, 500 rue JF Breton, Montpellier, FR-34093 France; 3 BBVA Data & Analytics, Avenida de Burgos 16D, Madrid, E-28036 Spain; 4 Instituto de Física Interdisciplinar y Sistemas Complejos IFISC (CSIC-UIB), Campus UIB, Palma de Mallorca, 07122 Spain

**Keywords:** Human mobility, Shopping mobility, Wealth inequality, Spatial networks, Graph rewiring

## Abstract

Socioeconomic inequalities in cities are embedded in space and result in neighborhood effects, whose harmful consequences have proved very hard to counterbalance efficiently by planning policies alone. Considering redistribution of money flows as a first step toward improved spatial equity, we study a bottom-up approach that would rely on a slight evolution of shopping mobility practices. Building on a database of anonymized card transactions in Madrid and Barcelona, we quantify the mobility effort required to reach a reference situation where commercial income is evenly shared among neighborhoods. The redirections of shopping trips preserve key properties of human mobility, including travel distances. Surprisingly, for both cities only a small fraction (∼5*%*) of trips need to be modified to reach equality situations, improving even other sustainability indicators. The method could be implemented in mobile applications that would assist individuals in reshaping their shopping practices, to promote the spatial redistribution of opportunities in the city.

**Electronic supplementary material:**

The online version of this article (doi:10.1007/s41109-017-0026-3) contains supplementary material, which is available to authorized users.

## Introduction

The growth of economic inequality has raised concern and attention in recent years (Piketty 2014; Stiglitz 2015). In cities these inequalities are embedded in space, as a result of entangled processes which include location choices of households and businesses, daily mobility, segregation and closure attitudes, central planning, or global economic restructuring. Over the course of several decades their joint actions have given rise to segregated cities, characterized by uneven distributions of capital among their neighborhoods. While the intensity of socioeconomic inequalities vary from one city to another, the general observation that “some neighborhoods are poorer than others” has been made for cities with different age, in every continent, and for different periods in urban history (Pinol 2003; Goldsmith et al. 2010; Cassiers and Kesteloot 2012; UN-HABITAT 2014). An abundant literature has long depicted the *neighborhood effect* (Friedrichs et al. 2003) – the neighbourhood impacts the life trajectories of the residents, even when controlling for their individual characteristics –, and highlighted its societal costs and enduring consequences (Blau and Blau 1982; Brooks-Gunn et al. 1997; Vallée et al. 2010; Womack 1972; Chetty and Hendren 2015).

Over the last decade, increasing volumes of digital geographic footprints have been produced by individuals using mobile ICT devices, and these footprints have been increasingly analyzed by scientists as well. These data are not free of biases (Lewis 2015) or privacy concerns (de Montjoye et al. 2015), but they undeniably constitute an important asset for understanding social phenomena in detailed spatio-temporal contexts (Lazer et al. 2009; Eagle et al. 2010; Onnela et al. 2007; Lu et al. 2012; Sun et al. 2013; González et al. 2008). They also have the potential to reveal the information required to coordinate individuals’ actions, so that large groups of people can tackle issues which are distributed and spatial by nature. This is particularly true in the case of mobility networks, which already integrate such footprints in feedback mechanisms: people produce data when moving, and their travel decisions are partly guided by the data produced by others. Examples include GPS navigation using real-time traffic data, local search and discovery of new places, or location-based dating applications. So far, these footprints have been mainly used in applications intended to enhance individual satisfaction (time savings, discovery of a location, encounter of a partner), but they have also fostered spontaneous and large-scale solidarity movements during disasters (e.g. Facebook’s safety check, or the use of dedicated Twitter hashtags). An important question is thus whether we can scale up, and address complex issues through distributed and coordinated approaches relying on such data. Here we refer to complex social issues for which improvements would necessarily occur on longer timescales. There is a need to relate smart technology with sustainability and spatial justice in cities (McLaren and Agyeman 2015), and this implies building upon the existing practices of individuals. In this work, we develop further this idea by focusing on a complex problem: the reduction of spatial inequality in large cities.

The “Robin-Hood effect” refers to a process through which capital is redistributed to reduce inequality. A spatial and city-scale implementation would then consist in taking from the rich neighborhoods to give to the poor. This role is normally played by the city’s governance, and is essential to mitigate spatial inequality. However, studies in cities worldwide have demonstrated that top-down planning and fiscal policies alone seem inefficient in significantly counterbalancing the numerous consequences of the neighborhood effect (Ostendorf et al. 2001; Satterthwaite and Mitlin 2013). It has also been long emphasized that developing economic activity in disadvantaged regions indirectly benefits the surrounding populations, by fostering job opportunities, transport facilities and increased safety (Kanbur and Venables 2005). Here, we study an original approach to rebalance economic activity among a city’s neighborhoods. The scenarios we explore would not incur any additional environmental or monetary costs, but would instead require slight modifications of daily shopping mobility practices.

According to surveys, shopping and leisure trips account for 15 to 20*%* of the individuals’ daily travels (AASHTO 2013). Such trips virtually move money from one part of the city to another, and directly contribute to shape the spatial distribution of wealth across neighborhoods. By connecting areas, shopping trips also foster metropolitan integration and “social cohesion” (Miciukiewicz and Vigar 2012), whilst the resulting money flows are a key component of the development of territories (Ruault 2014). Large metropolitan areas are characterized by mixed land use in many of their neighborhoods. Every time a resident has to buy usual products such as food, gas or clothes, he/she can actually indeed choose among several stores and neighborhoods to do so, sometimes without even increasing his/her travel time.

In the following we focus on the two largest Spanish cities, Madrid and Barcelona. Performing exploratory experiments, we demonstrate that they could be more evenly balanced thanks to the cumulative addition of small and reasonable changes in a limited fraction of their residents’ shopping destinations. While there exists various spatial indicators for quantifying territorial inequalities, static indicators fail to provide a clear picture of the collective effort that would be required to reach a certain level of redistribution. With this in mind, we quantify the proportion of individual shopping trips that should be redirected in order to evenly share the commercial income between neighbourhoods, the first step of a conceivable path toward a spatial redistribution of opportunities in the city. We show that alternative mobility scenarios not only allow to distribute money more evenly in space, but also to enhance the spatial mixing of residents through their shopping mobility, without increasing the total distance traveled, nor changing the individuals’ effective purchases and mobility routines. The following of this paper is an exploration on how money flows in cities could be more evenly distributed, if shopping spatial behavior was slightly restructured. In the following of this paper, we use zipcodes delimitations as proxies for neighbourhoods. While zipcodes correspond to administrative units which are very coarse-grained proxies for effective neighbourhoods, this choice was imposed by the spatial resolution of the available data.

## Material and Methods

### Data

**Fig. 1:**
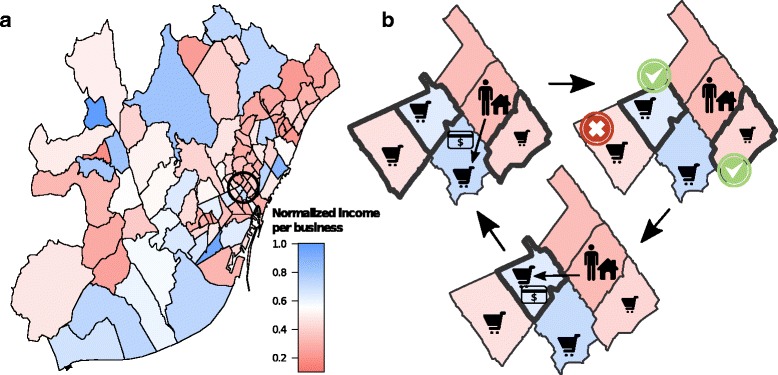
Rewiring urban shopping trips. **a** Average income per business in the neighborhoods of Barcelona resulting from individual transactions. The average income has been normalized by the maximum value among neighborhoods. The data correspond to 2011 and is displayed by zip code in the metropolitan area. From this perspective, some neighborhoods are five times richer than others. **b** The general principle common to the iterative rewiring methods. At each step a transaction is randomly selected, along with the possible alternative businesses (*highlighted in bold*). If rewiring the transaction to one of them (randomly selected) decreases inequality between neighborhoods and matches the other constraints, then rewiring is performed

We use a dataset containing the metadata of one year of bank card payments from more than 150,000 anonymous users in over 95,000 businesses of Barcelona and Madrid. Each transaction is time-stamped and contains the information collected by the bank on both the cardholder and the business. It also includes the customer’s age and residence’s zipcode, the business category and its geographical coordinates (see Additional file 1 for details and (Martinez et al. 2016; Yoshimura et al. 2016) for other recent examples of research relying on similar data). From these data there are two obvious ways to estimate inequality among neighborhoods: first, in measuring the income of their residents – indirectly estimated through the amount of money spent during the year; second, in measuring the income resulting from the commercial activity of businesses located in these neighborhoods. The latter is particularly interesting because it results from the spatial organization of shopping trips, which may be much easier to alter than any other type of daily trips, notably commuting. The average commercial income of businesses in Barcelona’s neighbourhoods, resulting from shopping trips, is mapped on Fig. 1 a. This map reveals that according to this measure, some neighborhoods are indeed five times richer than others.

### Rewiring the shopping trips networks

From the data for both cities we construct the bipartite spatial network whose nodes are individuals and businesses, and whose edges stand for transactions (see Additional file 1: Figure S2). We then perform rewiring experiments, in which randomly selected transactions are redirected toward alternative businesses of the same category, but located elsewhere in the city (Fig. 1 b). The rewiring methods we implemented operate directly at the level of individual transactions (see Additional file 1: Figure S2). A rewiring operation then consists in randomly selecting a transaction *t*_*r,b*_ (made by user *r* in business *b*), and an alternative business *b* ^′^≠*b*, such than *b* ^′^ and *b* are of the same category (see in Additional file 1 the details of the 16 business categories), but located in different neighborhoods. The rewiring occurs only if the change fulfills a number of constraints which are expressed at the city level. The calculation of these constraints are based on the candidate configuration of the shopping trips network (i.e. after *k*+1 rewiring operations), the current configuration (after *k* rewiring operations) and the original shopping trips network.

#### Four dimensions to assess the likelihood of the network’s configuration

We consider four dimensions to assess the different network configurations from an economic, social and environmental point of view. Since our main objective is to rebalance the distribution of commercial income among the neighborhoods, we first focus on the economic dimension. We denote by *W*_*k*_ the *wealth inequality among the city’s neighborhoods* after *k* rewiring operations. It is defined as the distance to a reference homogeneous situation, where the commercial income resulting from purchases would be equally shared among all neighborhoods. We have:

1 $$ W_{k} = \sum\limits_{i=1}^{N} \left(\overline{w}^{i}_{k}-w^{*}\right)^{2},  $$

where *N* is the number of neighborhoods, $\overline {w}^{i}_{k}$ is the average income of the businesses located in the neighborhood *i* after *k* rewiring operations, and *w* ^∗^ represents the wealth per neighborhood in the reference configuration where commercial income is evenly distributed across neighborhoods, such that

2 $$ w^{*}=\frac{1}{N}\sum\limits_{i=1}^{N} \overline{w}^{i}_{k}.  $$

Another important aspect is related to the social nature of mobility in the city that might prevent some neighborhoods from ghettoization. To measure to what extent individuals residing in various neighborhoods mix in the city space as a result of their travels, for each neighborhood *i* we count the number of times $\left (s^{i1}_{k},...,s^{iN}_{k}\right)$ the residents of *i* traveled to each of the *N* neighborhoods (*i* included), after *k* rewiring operations. Then, by averaging the vector of trips over all the neighborhoods, we compute a geographical diversity index *S*_*k*_ (after *k* rewiring operations),

3 $$ S_{k} = \frac{1}{N}\sum_{i=1}^{N} \sum_{j=1}^{N} \left(s^{ij}_{k}-s_{i}^{*}\right)^{2},   $$

where $s_{i}^{*}$ represents the homogeneous distribution of visits originating from *i* and in direction to all neighborhoods,

4 $$  s_{i}^{*}=\frac{1}{N} \sum_{j=1}^{N} s^{ij}_{k}.  $$

The third considered dimension is the distance traveled by individuals. Summing the distances traveled by individuals for all their shopping trips, we can compute *D*_*k*_ the total distance traveled, as measured after rewiring *k* transactions. Details about the method used to estimate the shopping trips distances are available in Additional file 1.

Finally, we are also interested in individual mobility routines and the tendency of individuals to return to already visited places. For each individual we calculate an exploration rate *ρ*_*k*_. It is defined as the number of unique businesses he/she has visited divided by his/her total number of transactions, after *k* rewiring operations. Considering the empirical peaked distribution of *ρ*_*k*_ among the population of customers (see Fig. 3 b), in the following we only consider the average value $\bar {\rho }_{k}$.

#### Rewiring constraints

As mentioned above, a candidate reconfiguration of the shopping trips network (*k*+1 rewiring operations) will occur if and only if the proposed change respects a number of constraints regarding the current configuration (*k* rewiring operations) and the original shopping trips network. We consider four constraints, each of them concerns one of the four economic, social and environmental dimensions described in the previous section,

- A first constraint applies on the *wealth distribution*; it ensures that each destination change contributes to iteratively homogenize the distribution of commercial income across neighborhoods.
- A constraint on the *spatial mixing* of individuals resulting from their shopping travels. In order to be accepted, a rewiring operation has to preserve the diversity of neighborhoods visited, hence the degree of spatial mixing of individuals residing in different neighborhoods.
- A third constraint on the *total distance traveled*, to guarantee that each destination change does not result in increasing the total distance traveled. The distance associated to each individual transaction is calculated with regard to the individual’s main activity place at this moment of the day.
- Finally, a constraint on the spatial *exploration rate* of individuals, to preserve the behavioral mobility routines measured in the population.

All constrains have the same form and are satisfied if the following condition holds

5 $$ X_{k+1} \leq\left\{ \begin{array}{ll} X_{k} & \text{if}~ X_{k+1} \geq \alpha X_{0},\\ \alpha X_{0} & \text{otherwise}, \end{array} \right.  $$

where *k* denotes the number of rewiring operation, and *α* is a parameter positive or equal to zero. The general form of Eq. 5 allows us to fix an objective upper bound for each dimension *X*_*k*_ with respect to its original value *X*_0_. Then as long as *X*_*k*_ is greater than *α* *X*_0_, each rewiring operation must decrease *X*. Once *X*_*k*_ is smaller than *α* *X*_0_, then rule 5 ensures that none of the following rewiring operations will increase *X*_*k*_ above *α* *X*_0_. An experiment is then defined by a set of four values $(\alpha _{W}, \alpha _{S}, \alpha _{D}, \alpha _{\bar {\rho }})$ that specify the maximal value desired for each variable of interest.

#### Algorithm

The process is unambiguously specified by Algorithm 1. One should note that the products purchased and the amount of expenses of each individual are preserved. This iterative process is run until the rewiring rate falls below a given threshold (see the Additional file 1 for more details). Since the rewiring process is stochastic, all the results we discuss in the following sections have been averaged over hundreds of replications. Numerous rewiring methods fulfilling the aforementionned conditions could be proposed. However, we favored a numerical approach because of the large number of transactions (∼10^7^) and also because of the constraints we impose to guarantee realistic and interesting properties.

Besides, the random selection of (i) the transaction to rewire and (ii) of the candidate business (step 1. of the algorithm) can be uniform (denoted “Uniform” sampling hereafter) or proportional to their amount in the case of transactions and/or inversely proportional to the average income of the targeted neighborhood for the businesses (denoted “Weighted” sampling hereafter). Even more informed methods might be proposed, but for the sake of simplicity only simple random procedures are tested in the following.


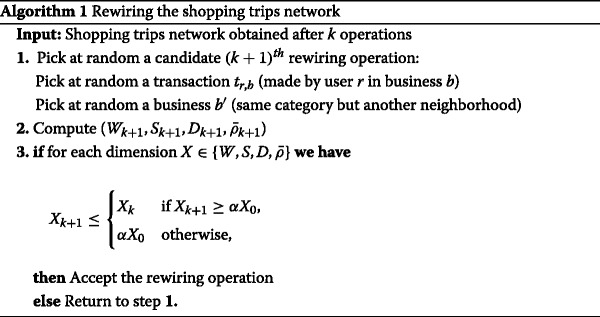


## Results

### Reachability of even spatial distributions

**Fig. 2:**
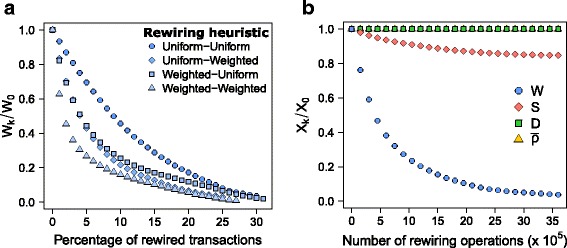
Decreasing spatial inequality in the city by adapting daily shopping destinations. **a** Decrease of wealth inequality among neighborhoods as a function of the fraction of transactions rewired, for various rewiring methods. Four combinations of choice heuristics are considered, “Uniform-Uniform”, “Uniform-Weighted”, “Weighted-Uniform” and “Weighted-Weighted”. **b** Decrease of wealth inequality (*W*_*k*_/*W*_0_) while preserving the spatial mixing index (*S*_*k*_/*S*_0_), the total distance traveled (*D*_*k*_/*D*_0_) and the exploration rate ($\bar {\rho }_{k}/\bar {\rho }_{0}$), as a function of the number of rewiring operations. Values have been averaged over hundreds of replications. The bars represent the minimum and the maximum values obtained but in most cases are too close to the average to be seen (see Additional file 1: Figure S10-S11 for Madrid)

We first investigate the reachability of an even spatial distribution of the commercial income resulting from individual purchases, while the variables *S*, *D* and $\bar {\rho }$ remain in the range of their empirical values. To address this question, we apply the rewiring method previously described with the four constraints of Eq. 5 such as *α*_*W*_=0, *α*_*S*_=1, *α*_*D*_=1 and *α*_*ρ*_=1. This constitutes our *Reference* scenario. Figure 2 a shows the evolution of inequality in the urban area of Barcelona as a function of the fraction of rewired transactions, according to various sampling methods. Surprisingly, even with basic random sampling methods, it is possible to reduce spatial inequality between neighbourhoods by more than 80*%* while reassigning only 20*%* of individual transactions. All the methods produce the same qualitative behavior – an early regime of very fast decay, followed by a regime of slower decay. Weighted methods are naturally more efficient, and allow to reach spatial equity by redirecting a smaller fraction of transactions. In particular, a reduction of wealth inequality of 80*%* (*W*_*k*_/*W*_0_) can be obtained by rewiring only 5*%* of the transactions if the sampling method is double weighted.

The state of the other variables *S*, *D* and *ρ* is also monitored along the process, as shown in Fig. 2 b for a Uniform-Uniform sampling method. What makes the previous results remarkable is in fact that income redistribution is achieved without increasing the distance traveled by individuals (*D*), nor changing their mobility routines ($\bar {\rho }$). Moreover, a positive side-effect is to increase the frequency of encounters of individuals living in different parts of the city – as indicated by the decrease of *S*_*k*_/*S*_0_ –, an effect that could not be anticipated from the rewiring constraints alone (*α*_*S*_=1). The increase of spatial mixing is the consequence of individual shopping trips more evenly distributed in the city space, required to homogenize the income among neighborhoods. The behavior of *S* is non trivial, notably because one could imagine unrealistic solutions that would simultaneously even the spatial distribution of business income and decrease the total distance traveled, by rewiring most of the shopping trips to the closest neighborhood containing businesses of the relevant category. In this case the spatial mixing of individuals would decrease dramatically, and *S* the distance to an homogeneous mixing would increase. Here the decay of *S*/*S*_0_ guarantees that it is not the case.

### Preservation of human mobility properties

We wish to control further the likelihood of the rewired shopping mobility networks, and ensure that they preserve the spatial properties of individual human mobility. A small set of indicators have proved to be useful to describe the statistical and spatial properties of human mobility (González et al. 2008). These include the jump length between consecutive locations *Δ*_*r*_, the radius of gyration *r*_*g*_ (with $r_{g}(i) = \sqrt {\frac {1}{N}\sum _{j=1}^{N}(x_{j}(i)-x^{*}(i))^{2}}$, *r*_*g*_(*i*) is the characteristic travel distance of individual *i* with center of mass *x* ^∗^(*i*) after *N* displacements), and the tendency to return to already visited places *ρ*. In our case, for each individual *ρ* is simply defined as the ratio between the number of unique businesses visited and the total number of transactions.

**Fig. 3:**
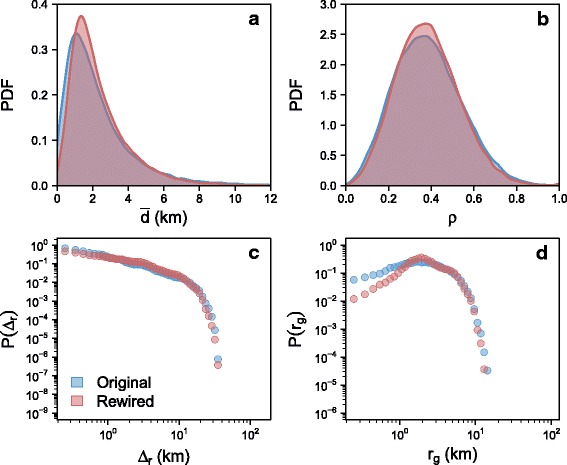
Observed and simulated distributions of human mobility indicators. The distribution of jump lengths *Δ*_*r*_ (**c**), the radius of gyration *r*_*g*_ (**d**), the tendency to return to already visited places (*ρ*) (**b**) and the individual average distance traveled ($\bar {d}$) (**a**) are considered. Values measured on the empirical data are in *blue*, while those obtained after rewiring are in *red*. The calculation of *Δ*_*r*_ and *r*_*g*_ is based on the business’ exact geographical coordinates. The simulated distributions plotted here correspond to one particular replication, see Additional file 1: Figure S4 for the robustness of the results and Additional file 1: Figure S12 for the same curves for Madrid

Figure 3 shows their empirical and simulated values, plus the average distance $\bar {d}$ traveled by each individual for each shopping trip (see the Additional file 1 for details on the calculation of shopping trips distances). On each panel both curves overlap almost perfectly, indicating that the rewiring has no significant effect on the key mobility properties. The simulated distributions of $\bar {d}$ and *r*_*g*_ are slightly more peaked than the empirical ones.

We showed in a previous study that young adults tend to spend their money further from their neighborhood of residence (Lenormand et al. 2015). Coherently their shopping trips are those that are the most affected in the simulated scenarios (see Additional file 1: Figure S6). But the simulated scenarios also contain some questionnable aspects. For example, elderly people are those whose displacements would increase the most (with regard to their current shopping travel distances – see Additional file 1: Figure S5).

### Multi-objective improvement

**Fig. 4:**
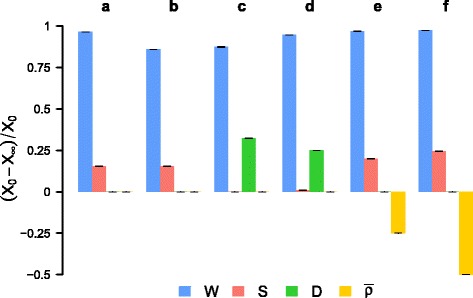
Multi-criteria improvement of shopping mobility. Each group of bars gives the relative gains or losses for the four indicators *W*, *S*, *D* and $\bar {\rho }$. Experiments are described in Table 1. See Additional file 1: Figure S13 for Madrid

We now perform multi-criteria rewiring experiments in order to measure to what extent redistribution can be achieved while improving simultaneously other important aspects of urban mobility. To this end, we perform the series of experiments summarized in Table 1. The objective is to even the wealth distribution among neighborhoods (*α*_*W*_=0) and also improve either *S*, *D* or $\bar {\rho }$ without worsening the other two. Figure 4 gives the relative gains and losses upon the four indicators, and the last two columns of Table 1 contain the asymptotic values obtained for the reduction rate of wealth inequality, for Barcelona (B) and Madrid (M).

**Table 1:** Experiments performed. Column W indicates the relative gain of (*W*_0_−*W*_*k*_)/*W*_0_. The first value is for Barcelona (B) and the second for Madrid (M)

Experiment	*α*_*W*_	*α*_*S*_	*α*_*D*_	$\alpha _{\bar {\rho }}$	W (B/M)
(a) Reference	0	1	1	1	96.4*%*/99.5*%*
(b) Spatial mixing *↑*	0	0.75	1	1	85.9*%*/78.1*%*
(c) 50*%* energy savings	0	1	0.5	1	87.4*%*/84.8*%*
(d) 25*%* energy savings	0	1	0.75	1	94.7*%*/98.8*%*
(e) Exploration rate *↑*	0	1	1	1.25	96.8*%*/99.9*%*
(f) Exploration rate *↑* *↑*	0	1	1	1.5	97.3*%*/100*%*

These experiments proove that it is not always possible to combine significant improvements on several dimensions simultaneously. This is not an issue with the method, but rather with the set of objectives which are somewhat opposite. Most individuals perform their shopping trips near their residence – as highlighted by the empirical distributions in Fig. 3 – and consequently it is not feasible to diversify the neighborhoods where an individual regularly travels to – in order to improve spatial mixing *S* – and at the same time decrease the total travel distance *D*. More surprisingly, experiment (b) indicates that it is also not possible to simultaneously improve the wealth redistribution and the spatial mixing of individuals. The two indicators are based on different metrics (the amount of money spent per business for *W* and the number of trips for *S*), which imply different reference egalitarian situations (see Additional file 1: Figure S7 for more details). Optimization is thus a trade-off between the various consequences of shopping mobility at the city scale. However, experiments (c) and (d) prove that it is possible to significantly decrease the total distance traveled and in the same time to strongly reduce wealth inequality among neighborhoods, but not as much as in the reference experiment. Still, it is remarkable that experiment (d) results in an alternative mobility network such that the spatial inequality of the average business income is reduced by 95*%*, while the total distance associated to shopping mobility is reduced by 25*%*, the level of spatial mixing is preserved, as well as the individual mobility routines. Finally, experiments (e) and (f) show that even if residents deeply restructured their mobility routines, and typically started going to a new business each time they perform a new shopping trip, keeping control of the total distance traveled in the city would prevent from increasing the mixing of individuals coming from different neighborhoods beyond 25*%*. The gains in terms of wealth redistribution would not be significant when compared to the reference experiment (a).

## Discussion

Reducing urban segregation and increasing spatial justice are some of the major challenges faced by cities worldwide, and the digital footprints passively produced by their residents constitute a promising resource to help addressing these issues from the bottom. This study is a first attempt to quantify the relation between shopping mobility and the spatial distribution of economic activity in the city. The alternative shopping trips resulting from our experiments offer an interesting trade-off between the preservation of essential aspects – the effective purchases of individuals and households, and their mobility properties – and some reasonable changes in the places where they spend their money. The addition of small changes in the shopping destinations of individuals can dramatically impact the spatial distribution of money flows in the city, and the frequency of encounters between residents of different neighborhoods, even if the total number of changes remains relatively small. These results have important consequences, and they lead in particular to the decisive question of the effective implementation of alternative shopping travels, like those drawn by our experiments. While the decision process behind each individual redirection may appear intricate for a single person, one could easily imagine dedicated mobile applications, querying databases similar to the one we used in this paper. Their purpose would be to assist their users in a transition toward a more socially and spatially concerned shopping mobility.

### Limitations of the study

However, one should keep in mind that individuals do not guide most of their travel decisions by philanthropy, but instead by balancing accessibility, price and business characteristics. Individuals first choose their casual shopping destinations with regard of transport facilities and travel time budget (Recker and Kostyniuk 1978). Here as accessibility information we considered the Euclidean distance between neighborhoods. However, in urban environments the Euclidean distance is rarely a direct proxy for travel time (Gallotti et al. 2016), and some of the rewired shopping trips are unlikely to be performed in the real world. more accurate measures of travel time. In future studies transport APIs and road network data could be used instead to calculate more realistic travel distances between points in the city. Also, from a choice point of view, distance is not the only one determinant of shopping destination choices. The inequality might be also due to the inequality between businesses of the same category, which may differ substantially in terms of the price, product quality, etc. The reader should also remind that prices of retail goods are not uniform across a city’s neighborhoods, they may display strong spatial variation, affecting consumer choices and spatial behavior in complex ways.

We also assumed that every shopping trip follows the simple pattern *A*→*B*→*A*, and we did not consider the more complicated case of chained trips (e.g. *A*→*B*→*C*→*A*) during which individuals join several trips associated with different purposes (Schneider et al. 2013). Destination choices for certain types of shopping travels are also motivated by reasons that are not only related to the sole proximity (e.g. the local market is a place where residents can build a sense of community, discuss the problems of the neighbourhood, make social contacts useful in everyday life). People also tend to choose the places where they spend money according to several other key factors, the price of products in the first place, but also according to some more personal appreciations, such as the "atmosphere" of neighborhoods and the feeling of well-being they provide. In large cities, the neighborhoods strongly differ in the quality of their planning and architecture, in their public spaces, in their amenities and leisure opportunities, commercial fabric, in their safety. Additionally, the changes might be considered as problematic, since a profound spatial reorganization of shopping mobility in the city could have consequences on the spatial structure of employment in the first place (see Additional file 1: Figures S8 and S9 showing the evolution of the number of clients and transactions per business), then on residences, finally on their commercial offer and ambience. This questions the likelihood and desirability of the objective configuration we chose, in which the average commercial income per business is evenly balanced across neighbourhoods. Still it has the advantage to be unambiguously defined, and constitutes an immediate, easy-to-think-with reference situation

For the sake of simplicity, we considered none of the aforementionned factors but they could be implemented in more involved frameworks derived from our work. A number of additional constraints, aiming at making the rewiring schemes more realistic from a spatial economy perspective, could be introduced in future scenarios. These include the preservation of the number of transactions and income per business (or at least a certain fraction of them); the restriction of the rewiring operations to certain business categories; limiting changes for certain socio-demographic categories of population (such as elderly people); or ensuring the temporal likelihood of the simulated scenarios (so that the rerouted shopping travels are homogeneously distributed through time).

### Concluding remarks

There are recent, encouraging examples of fast and wide adoptions of new daily practices, whose benefits are essentially collective. Examples include garbage differentiation (Robinson and Read 2005), the increasing role of bicycle in urban transport and the development of bicycle sharing systems (Dill and Carr 2003), or the open-source movement and the dedication of a growing number of individuals to collectively build free knowledge databases (e.g. Wikipedia, StackExchange, the free software movement). In these cases, the remuneration of participants, if any, is essentially symbolic. The success of a spatial counterpart to these altruistic behaviors could rejuvenate the very meaning of the so-called sharing economy (McLaren and Agyeman 2015). As citizens produce the data that document their location and activity patterns, in return these data could serve not only the specific interest of the institution collecting them, but also support fair socio-economic initiatives. Our study brings evidence that these geographical footprints we passively produce can support bottom-up responses to big societal issues, an expected feature of truly smart cities.

## Additional file

Additional file 1 Supplementary Information for Crowdsourcing the Robin Hood effect in cities. (PDF 782 kb)
