# Supplementary material for: Crowdsourcing the Robin Hood effect in cities
Source: Appl Netw Sci. 2017 Jun 8;2(1):11. doi: 10.1007/s41109-017-0026-3 (PMC6214245; doi:10.1007/s41109-017-0026-3)
Supplement: Supplementary file 1 — Supplementary Information for Crowdsourcing the Robin Hood effect in cities. (PDF 782 kb) [file 41109_2017_26_MOESM1_ESM.pdf]

Supplementary Information for
Crowdsourcing the Robin Hood effect in cities

Thomas Louail[1,2], Maxime Lenormand[3], Juan Murillo Arias[4], and José J. Ramasco[2]

[1]CNRS, UMR Gographie-Cits, 13 rue du four, FR-75006 Paris, France
[2]Instituto de Física Interdisciplinar y Sistemas Complejos IFISC (CSIC-UIB), Campus UIB, 07122 Palma de Mallorca, Spain
[3]Irstea, UMR TETIS, 500 rue JF Breton, FR-34093 Montpellier, France
[4]BBVA Data & Analytics,Avenida de Burgos 16D, E-28036 Madrid, Spain

Data preprocessing

The dataset contains information about 14 million bank card transactions made by customers of the Banco Bilbao Vizcaya Argentaria (BBVA) in the metropolitan areas of Barcelona and Madrid in 2011. For both case studies, we only consider the credit card payments whose amount was inferior to 1000 euros, and which were made inside the metropolitan areas, by bank customers that lived and worked in the metropolitan area in 2011. Each transaction is characterized by its amount (in euro currency) and the time when the transaction has occurred. Each transaction is also linked to a customer and a business. Customers are identified with an anonymized customer ID, connected with sociodemographic characteristics (gender, age and occupation) and their postcode of residence. In the same way, businesses are identified through an anonymized business ID, a business category id, and the geographical coordinates of the credit card terminal. Since we are primarily interested in daily shopping mobility, we chose to consider the business categories that account for the top 90% of the daily shopping trips (see Figure S1). The proportions of shopping trips associated to each of the 20 business categories we selected are available in Table S1.

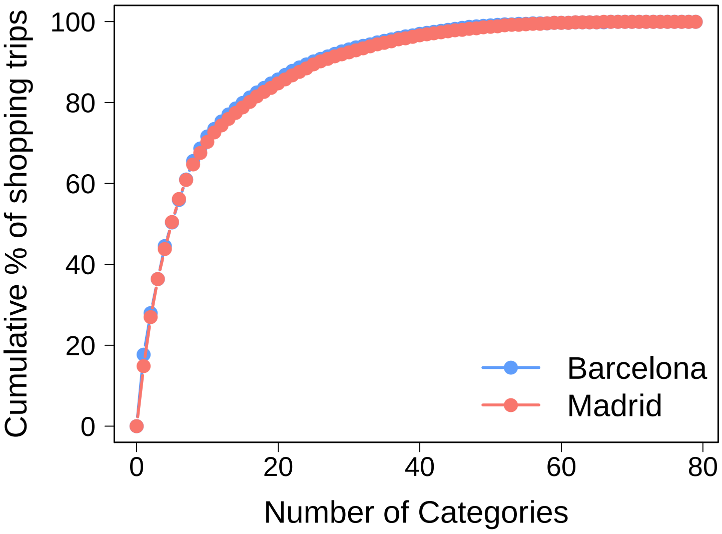

Figure S1: **Cumulative proportion of shopping trips as a function of the number of categories.** In blue the metropolitan area of Barcelona; In red the one of Madrid

Table S1: **Proportion of shopping trips associated to each of the 20 business categories selected.**

Category	Barcelona	Madrid
Supermarket	17.71	14.84
Hypermarket	10.25	12.09
Gas Stations	8.41	9.49
Restaurants	8.20	6.73
Retail store	5.84	2.82
Clothing store chain	5.48	4.67
Clothing store	5.16	7.33
Pharmacy, optical and orthopedics	4.52	3.81
Department store	3.14	5.73
Hair and beauty	2.88	2.72
Electronics, computers and appliances	1.97	1.44
Bars and café	1.85	1.60
Shoe store	1.71	1.43
Toys and sports articles	1.43	1.33
Bookshop, music shop and stationery	1.42	1.04
Fast food restaurants and chains	1.13	2.38
Car dealership and garage	1.02	1.01
Bazaar	1.01	1.06
DIY store	0.99	1.08
Hospitals, clinics and doctors	0.91	0.88

Formal description of the rewiring process

From the data we extract $G(R, B, T)$ the bipartite network of all credit card transactions performed by the city residents in businesses located in the city, during the entire year. R is the set of residents, B the set of businesses and T the set of transactions. Table S2 contains the characteristic attributes of the network in the two cities studied. Each city is partitioned in N spatial units/neighborhoods (here the units correspond to zip codes) and the network G is spatial: each resident and each business is located in one neighborhood. We denote R_i (resp. B_i) the set of residents (resp. businesses) located in the neighborhood i. The sets are disjointed and we have $R = \uplus R_i$ and $B = \uplus B_i$, with $i \in 1..N$. Additionally the businesses are also partitioned in C categories according to the products they sell, and we have $B = \uplus B_c, c \in 1..C$. The edges of the network represent the card transactions, hence implicitly the shopping trips. We note $t^k_{r,b}$ the k-th transaction performed by resident r in business b, and by $w(t^k_{r,b})$ its amount.

Table S2: **Summary statistics of the two metropolitan areas and of the two transactions networks.**

Statistics	Barcelona	Madrid
Number of neighborhoods	97	123
Number of inhabitants (2009)	3,218,071	5,512,495
Area (km^2)	634	1,935
Number of customers	42,023	118,447
Number of businesses	40,618	55,148
Number of transactions	3,640,961	10,025,642

The rewiring methods we implemented operate directly at the level of individual transactions. Each

rewiring operation $t_{r,b} \rightarrow t_{r,b'}$ consists in selecting a business $b' \neq b$, such than b' and b are of the same category c, but located in different neighborhoods. The rewiring occurs only if b' fulfills a number of additional constraints (namely which are expressed at the level of the entire city.

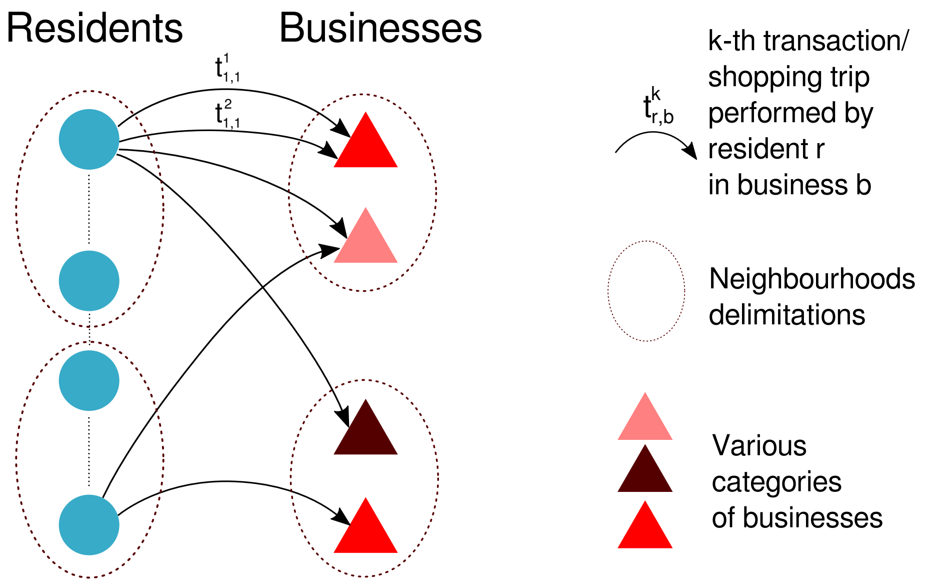

Figure S2: **The bipartite network of transactions.**

The network is rewired iteratively, i.e. transaction per transaction. A transaction $t_{r,b}$ is randomly selected. Then a neighborhood is selected among the set of all neighborhoods which contain businesses b' of the same category than b. Both the transaction and the candidate business can be picked up through a uniform or weighted random sampling ((the sampling may be uniform or weighted, we tested several sampling methods and compared their performance). If the rewiring operation $j \rightarrow j'$ matches the four constraints (C_W, C_D, C_S and C_ρ), then the transaction/edge is rerouted. The method stops when the rewiring rate falls below 0.001 (by convention).

Estimation of shopping trips distances and identification of the users' main daytime activity location

We made the assumption that to each transaction is implicitly associated a trip originating from either the main activity neighborhood during working time, or the neighborhood of residence, depending on the hour of the day and day of the week. The shopping trip distance is then defined as the Euclidean distance between the centroids of the origin and destination neighborhoods. We already know the neighborhood of residence that we can assign as the place of main activity during night time (i.e between 7pm and 8am) on week days and Saturday and Sunday. In addition to the neighborhood of residence, for each individual we can determine the neighborhood in which he/she was the most frequently located during the typical working hours of working days, i.e. from 8am to 7pm, from Monday to Friday. To do so, for each individual we count the number of unique couples $(day, hour)$ during which he/she was located in each neighborhood. For our study we keep only the individuals for which credit card is a casual mode of payment, and for which we can then reasonably assume that their card purchases and corresponding shopping trips are representative of their shopping mobility in general. Regarding the available statistics for Spain on the share of credit card payments among all payments, we decided to keep individuals whose data displayed at least $N = 20$ unique couples $(day, hour)$ during the entire year. For each of these individuals, we then determine the neighborhood in which they were the most frequently located during typical working hours. If this neighborhood accounted for less than one third of the time $\delta = 1/3$ in his/her entire set of locations, then the individual is discarded. As it can be seen

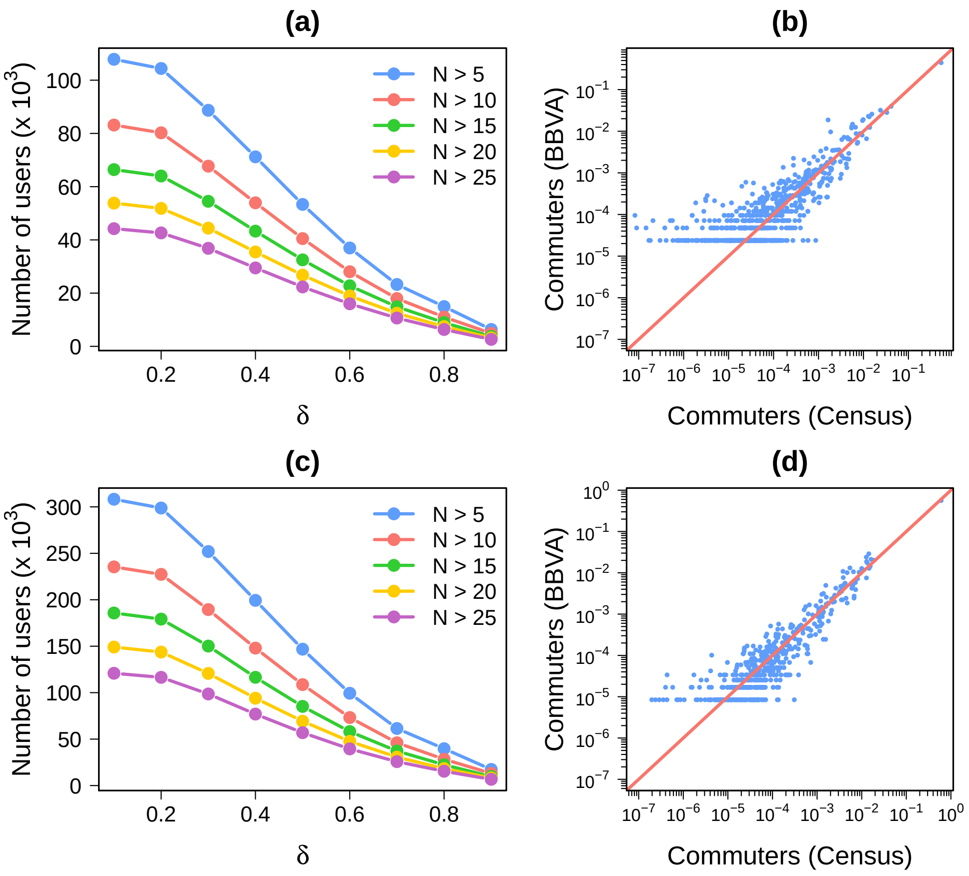

Figure S3: **Identification of the users' main daytime activity location in Barcelona ((a)-(b)) and Madrid ((c)-(d)).** (a) and (c) Number of users according to N and δ. (b) and (d) Comparison between the non-zero flows obtained with the credit card dataset ($(N, \delta) = (20, 1/3)$) and the census data. The values have been aggregated at the municipality scale. The values have been normalized by the total number of commuters for both OD tables. Blue points are scatter plot for each pair of municipalities. The red line represents the x = y line.

in Figure S3a and Figure S3c, the couple of value $(N, \delta) = (20, 1/3)$ allow us to keep enough users and discard the users not showing enough regularity to estimate their main daytime activity location. Finally, we can estimate the commuting flows between neighborhoods and assess the accuracy of the results by comparing these flows with those obtained from the 2011 Spanish census in Barcelona and Madrid [1]. The census data is at the municipal level, which implies that the neighborhoods must be aggregated at the municipality scale to be able to perform the comparative analysis. Figure S3b and Figure S3d show a scattered plot with the comparison between the flows obtained with the two matrices. A good agreement between the two ODs is obtained. The source code of this method is available at https://github.com/maximelenormand/Most-frequented-locations.

[1] Instituto Nacional de Estadstica (National Institute for Statistics). Available: http://www.ine.es. Accessed 2016 April 26.

Supplementary figures

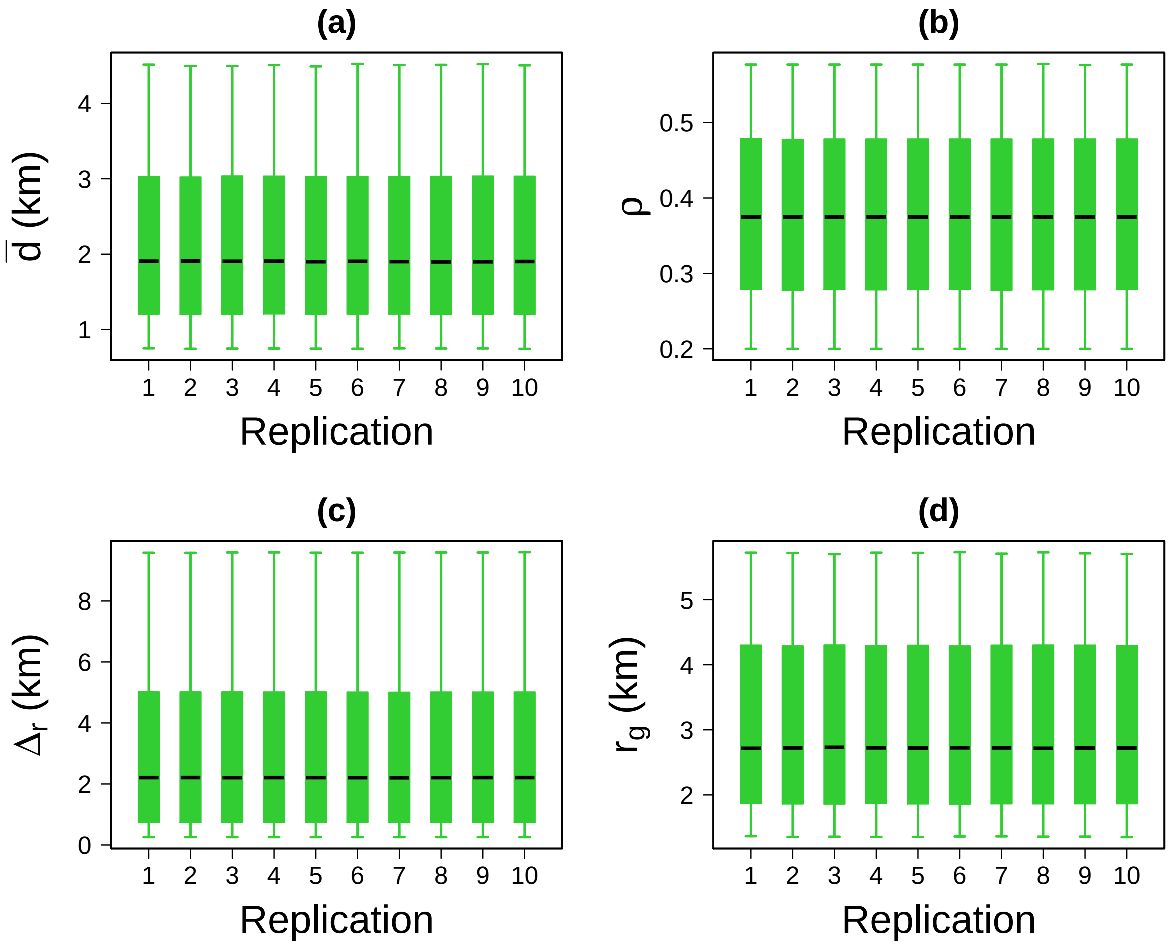

Figure S4: **Individual human mobility indicators' distributions obtained with ten replications of the algorithm.** (a) Individual average distance traveled $\bar{d}$. (b) Exploration rate ρ. (c) Jump length distribution Δ_r. (d) Radius of gyration r_g. The boxplot is composed of the first decile, the lower hinge, the median, the upper hinge and the 9th decile.

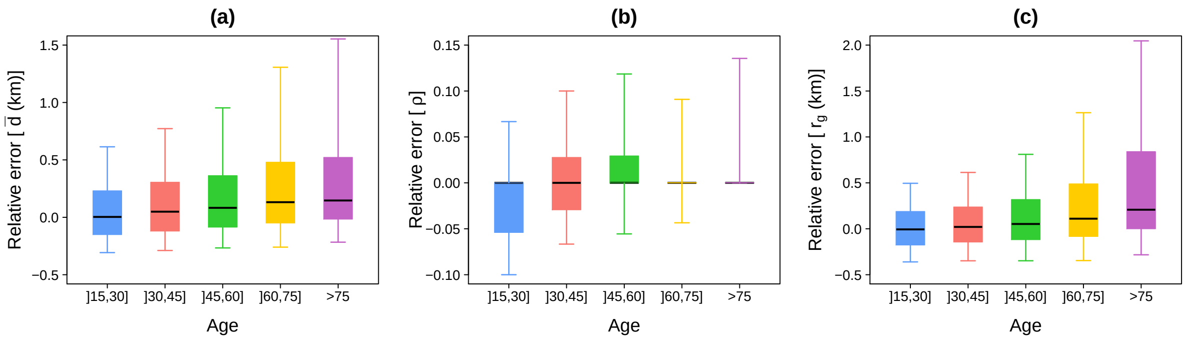

Figure S5: **Relative error between the original users' mobility indicators and the ones obtained after the rewiring according to the age.** (a) Individual average distance traveled $\bar{d}$. (b) Exploration rate ρ. (c) Radius of gyration r_g. The relative error is equal to the ratio of the difference between rewiring and original values and the original value. The boxplot is composed of the first decile, the lower hinge, the median, the upper hinge and the 9th decile.

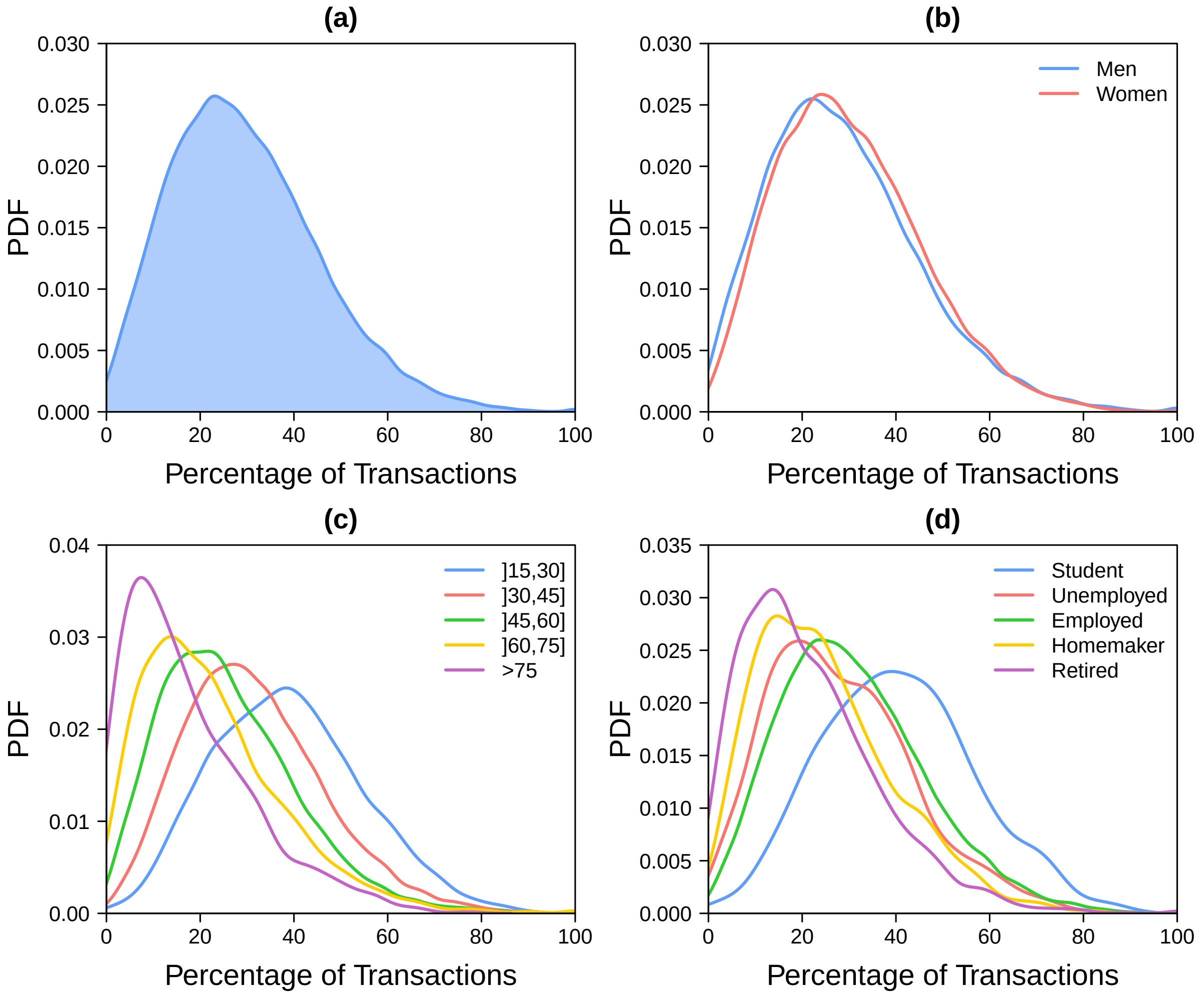

Figure S6: **Probability density functions of the individual percentage of rewired transactions.** (a) Total. (b) By Gender. (c) By age. (d) By occupation.

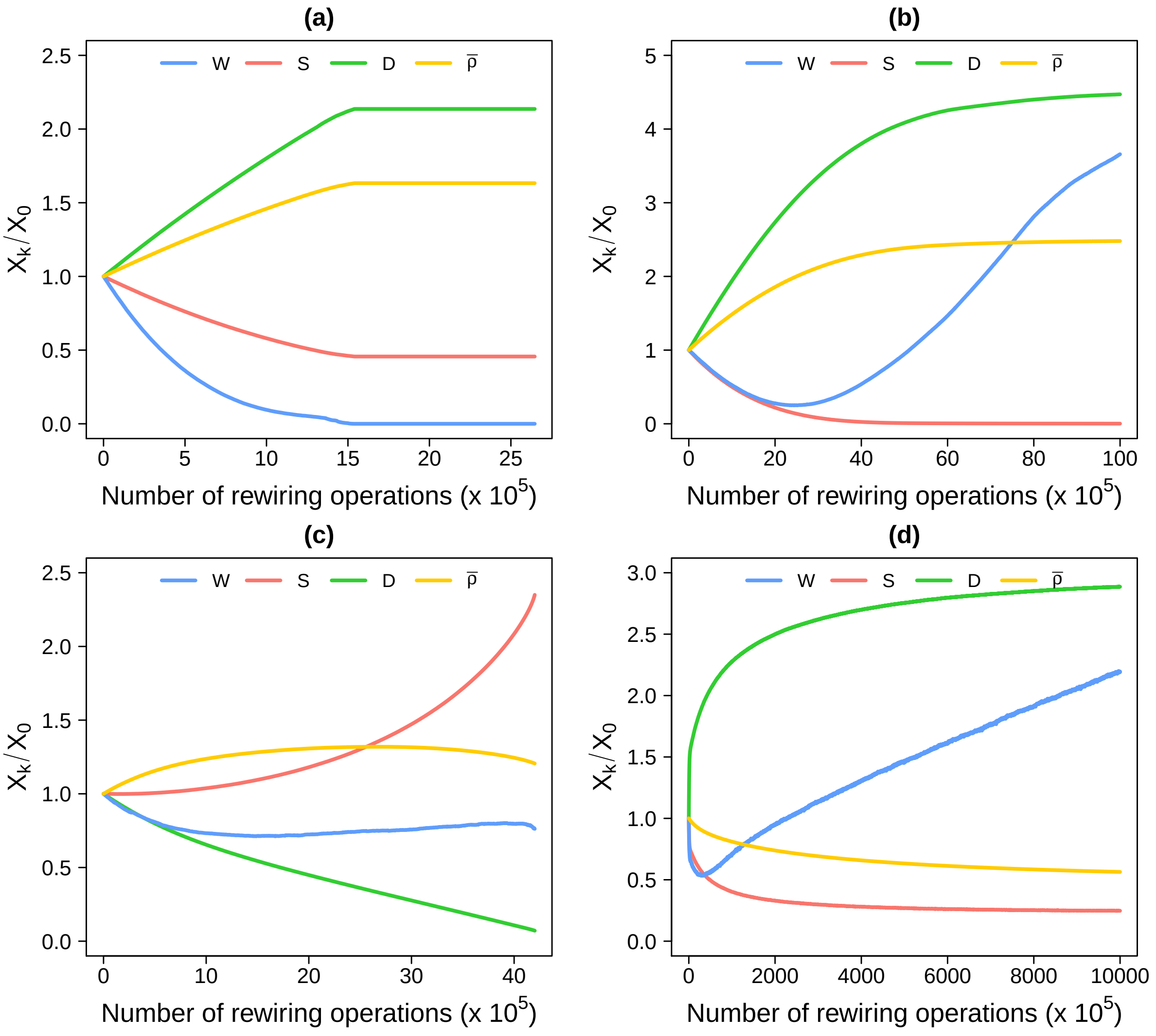

Figure S7: **Evolution of W, S, D and $\bar{\rho}$ as a function of the number of rewiring transactions.** (a) $(\alpha_W, \alpha_S, \alpha_D, \alpha_{\bar{\rho}}) = (0, +\infty, +\infty, +\infty)$. (b) $(\alpha_W, \alpha_S, \alpha_D, \alpha_{\bar{\rho}}) = (+\infty, 0, +\infty, +\infty)$. (c) $(\alpha_W, \alpha_S, \alpha_D, \alpha_{\bar{\rho}}) = (+\infty, +\infty, 0, +\infty)$. (d) $(\alpha_W, \alpha_S, \alpha_D, \alpha_{\bar{\rho}}) = (+\infty, +\infty, +\infty, 0)$.

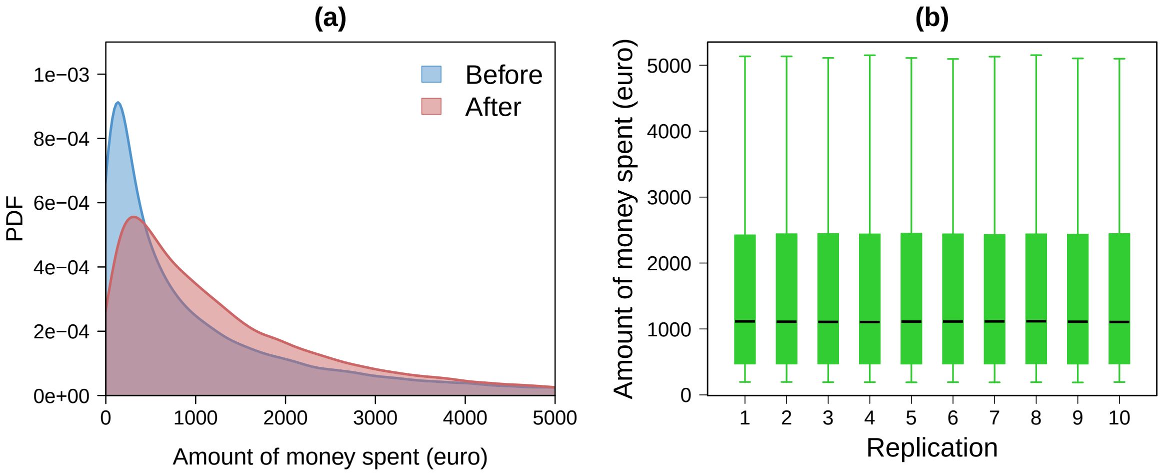

Figure S8: **Probability density functions of the total amount of money spent by business in 2011, in Barcelona.** (a) Comparison between the original distribution and the one obtained after applying the rewiring algorithm. (b) Distributions obtained with ten replications of the algorithm. The boxplot is composed of the first decile, the lower hinge, the median, the upper hinge and the 9th decile.

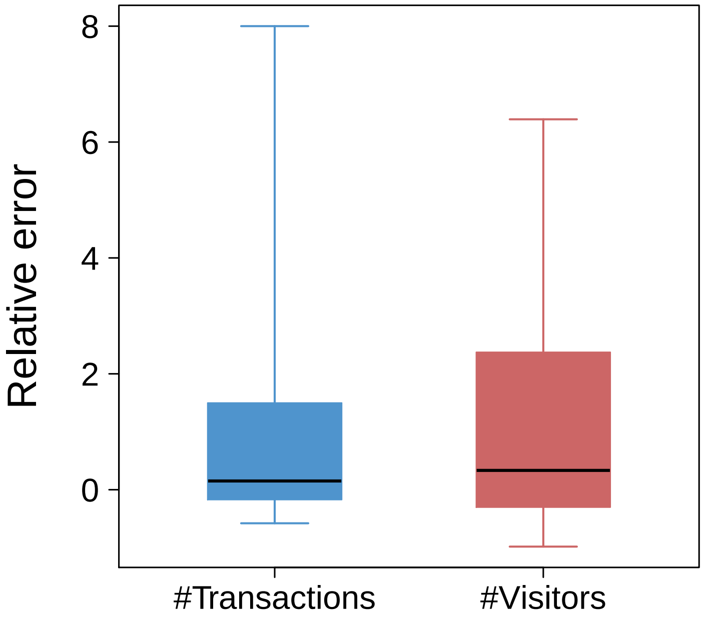

Figure S9: **Relative error between the original number of transactions and visitors and the ones obtained after the rewiring for each business.** The relative error is equal to the ratio of the difference between rewiring and original values and the original value. The boxplot is composed of the first decile, the lower hinge, the median, the upper hinge and the 9th decile.

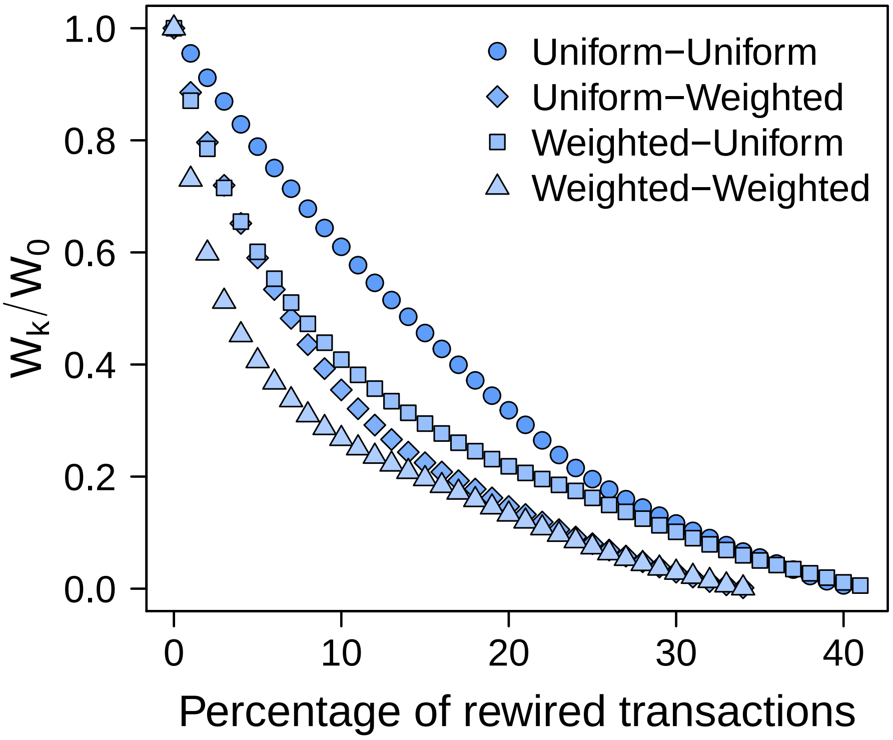

Figure S10: **Decrease of wealth inequality among neighborhoods as a function of the fraction of transactions rewired, for various heuristics (Madrid).** Four heuristics are considered, ”Uniform-Uniform”, ”Uniform-Weighted”, ”Weighted-Uniform” and ”Weighted-Weighted”.

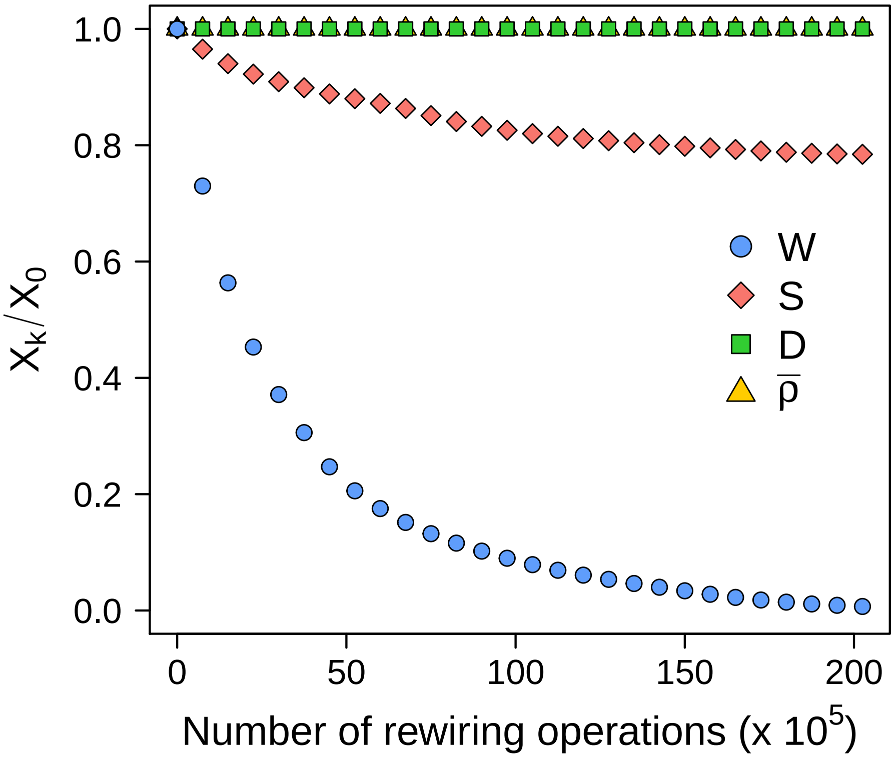

Figure S11: **Decrease of wealth inequality (W_k/W_0) while preserving the spatial mixing index (S_k/S_0), the distance traveled (D_k/D_0) and the exploration rate ($\bar{\rho}_k/\bar{\rho}_0$) as a function of the number of rewiring operations (Madrid case).** Values have been averaged over hundreds of replications. The bars represent the minimum and the maximum values obtained but in most cases they are too close to the average to be seen.

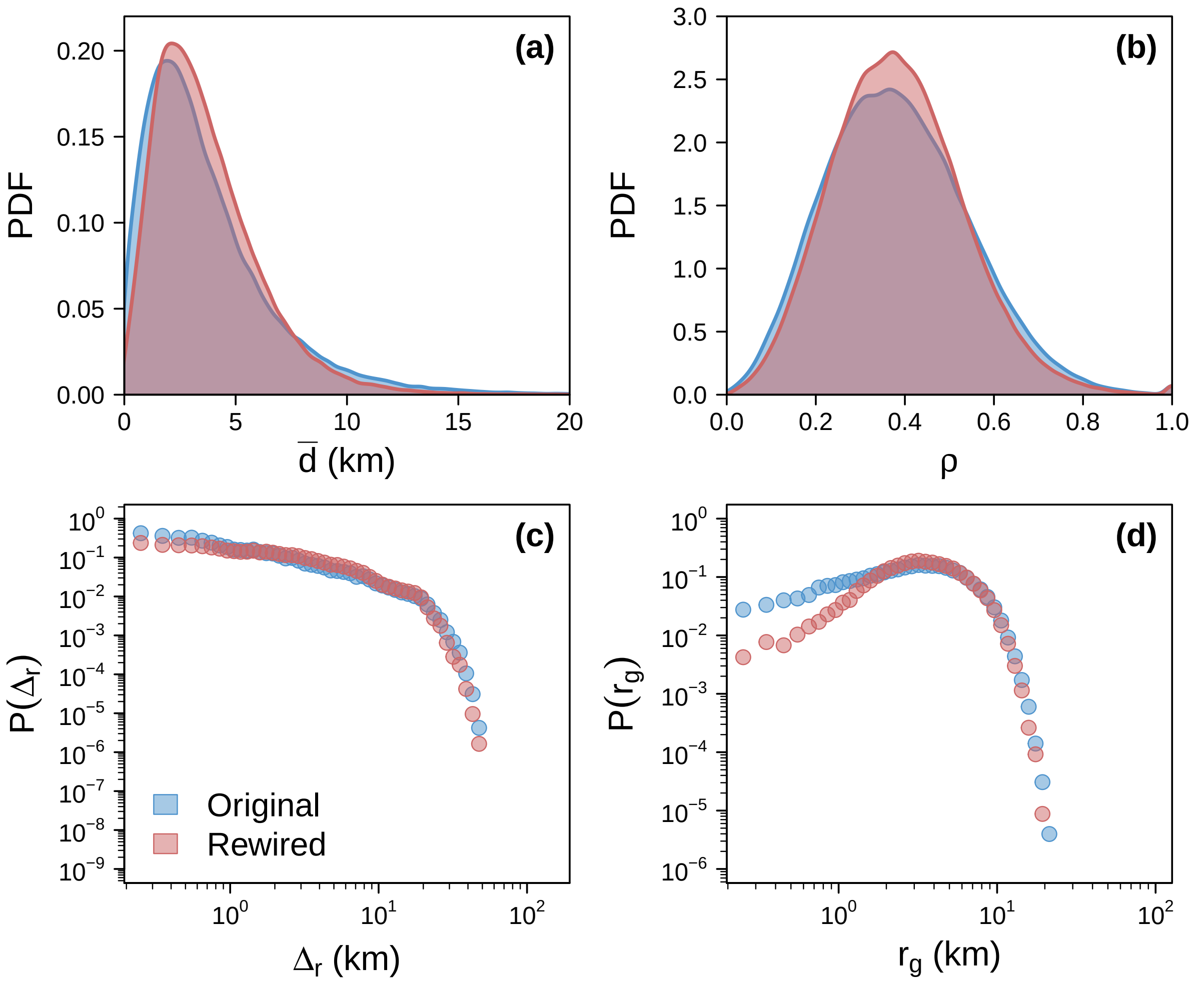

Figure S12: **Observed and simulated distributions of human mobility indicators in Madrid.** The distribution of jump lengths Δ_r, the radius of gyration r_g, the tendency to return to already visited places (ρ) and the individual average distance traveled ($\bar{d}$) are considered. Values measured on the empirical data are in blue, while those obtained after rewiring are in red. The calculation of Δ_r and r_g is based on the business' exact geographical coordinates.

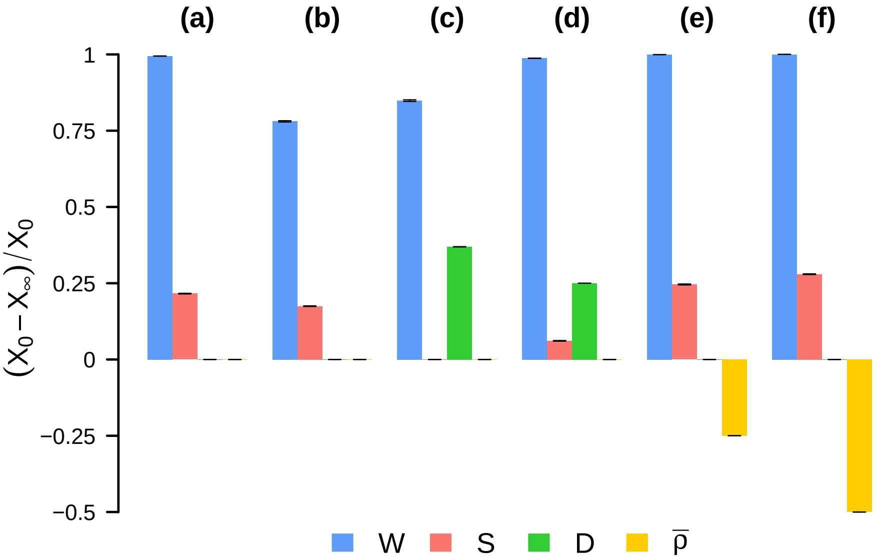

Figure S13: **Multi-criteria improvement of shopping mobility in the city of Madrid.** Each group of bars gives the relative gains or losses for the four indicators W, S, D and $\bar{\rho}$.
